# Relationship between laparoscopic and microscopic findings of peritoneum in peritoneal dialysis patients

**DOI:** 10.1007/s10047-022-01344-1

**Published:** 2022-08-03

**Authors:** Chieko Hamada

**Affiliations:** 1 Faculty of Health Science and Nursing, Juntendo University, Tokyo, Japan; 2 Faculty of Health Science and Nursing, Juntendo University, 3-7-33 Omiya, Mishima, Shizuoka 411-8787 Japan

**Keywords:** Peritoneal dialysis, Peritoneal injury, Laparoscopic findings, Microscopic findings, Morphology

## Abstract

Long-term exposure to the peritoneal dialysis solution (PDS) causes functional and morphological alterations that diminish the efficacy of peritoneal dialysis (PD). Macroscopic and microscopic findings, submesothelial compact zone (SMC) thickness and vascular patency, were associated with PD duration. The relationship between microscopic and laparoscopic morphological findings in PD patients was determined. A total of 78 laparoscopic intraperitoneal findings were recorded during PD catheter removal and 45 peritoneal tissues were obtained from the anterior parietal peritoneum. We examined macroscopic morphological findings in both parietal and visceral peritoneums and bowel movement and assessed the score semiquantitatively. SMC thickness and vascular patency were examined as microscopic findings. Total laparoscopic finding’s score (LFS) and microscopic findings, SMC thickness and vascular patency, were associated with PD duration. Total LFS was related to SMC thickness in both visceral and parietal peritoneum, whereas it was related to vascular patency in parietal but not in visceral peritoneum. There was no relationship between microscopic findings and peritoneal surface color, properties, vasculopathy, and adhesion. Total LFS in patients with newly formed membrane and omentum atrophy was higher than in those without. There was a significant relationship between microscopic and laparoscopic findings in PD patients. It is important to evaluate laparoscopic findings in more PD patients to find the predictive findings of encapsulating peritoneal sclerosis development.

## Introduction

Peritoneal dialysis (PD) is an attractive treatment option for end-stage kidney disease. Long-term exposure to the peritoneal dialysis solution (PDS) causes functional and morphological alterations that diminish the efficacy of PD [1–4].

Characteristic pathological findings of the abdominal parietal peritoneum in PD patients are shedding of mesothelial cells, submesothelial compact zone (SMC) thickening, occlusion of capillaries and venules, and angiogenesis [3, 5, 6]. SMC thickening has already been observed in uremic condition and it progresses during PD period, and SMC in PD patients with ultrafiltration failure is significantly thicker than other cases [3]. Vascular lesions progress from vascular wall thickening during PD period and vascular patency rate decreases [3, 4]. Furthermore, vascular patency is reduced using a neutral PDS compared with a conventional acidic PDS [7–9]. Peritoneal injury due to PD treatment is not only limited to functional impairment, but also 0.7–7.3% of PD patients progress to encapsulating peritoneal sclerosis (EPS), a fatal complication [10, 11]. In EPS, bowel obstructive symptoms develop due to encapsulation of the adhered intestinal tract, forming a cocoon. In a multicenter study in Japan, 48 of 1958 PD patients progressed to EPS, and about 70% developed after PD discontinuation [12]. However, characteristic peritoneal tissue injury that can predict the onset of EPS after PD discontinuation has not yet been determined.

It is important to establish an evaluation of the changes in PD patients and strategies for preventing peritoneal injury and EPS development. We performed removal of the PD catheter using a laparoscope when PD was discontinued and, at the same time, observed the abdominal cavity to evaluate peritoneal injury due to PD, including formation of a new capsule. This study showed that laparoscopic findings showed a variety of findings over the PD period and that both findings of the abdominal parietal and visceral wall increased with a positive correlation with the PD period [13].

There is still no study on the relationship between macroscopic and microscopic findings in injured peritoneum due to PD. In addition, there is no examination of macroscopic findings for predicting EPS. In this study, we determined the relationship between microscopic and macroscopic morphological findings in the peritoneum of PD patients and examined the significance of laparoscopic findings in predicting EPS.

## Patients and methods

### Patients

Laparoscopic findings were recorded during PD catheter removal. Statement of Ethics.

The study was conducted at Juntendo University Hospital from 2002 to 2017 and approved by the Ethics Committee. Before conducting the study, the author and colleagues gave the subjects written informed consent approved by the Hospital Ethics Committee. The research was conducted ethically in accordance with the World Medical Association Declaration of Helsinki. We conducted this study with written consent from individual cases. Cases that had peritonitis within 1 month were excluded because of the effects of acute injury to the peritoneal tissue. Seventy-eight PD patients including 5 patients with diabetic nephropathy were enrolled in this study (Table 1). Average duration of PD was 85.4 ± 49.5 months. Forty-nine patients had used acidic PD fluid during PD treatment. Twenty-four patients had peritonitis, and 3 patients discontinued PD after improved episode because of persistent peritonitis. Hybrid therapy, a combination therapy of PD and once a week hemodialysis, was performed in 33 patients. Peritoneal lavage or rest after PD discontinuation until PD catheter removal was performed in 58 patients. Seven patients developed EPS after PD discontinuation. Causes of PD discontinuation were transition to planned HD in 66 cases, ultrafiltration failure in 4 cases, uremia in 3 cases, and others in 5 cases.

**Table 1:** Patients

Gender	(Male: female)	58:20
Age at the start of PD	(Cases)	48.6 ± 13.0
DM	(Patient)	5
Duration of PD	(Months)	85.4 ± 49.5
Use of acidic PD fluid	(Cases)	49
Episode of peritonitis	(Cases)	24
Hybrid therapy	(Cases)	33
Peritoneal lavage and/or rest	(Cases)	58
EPS	(Cases)	7
*Causes of PD discontinuation*		
Transition to planned HD	(Cases)	66
UFF	(Cases)	4
Uremia	(Cases)	3
Others	(Cases)	5

*PD* peritoneal dialysis, *DM* diabetes mellitus, *EPS* encapsulating peritoneal sclerosis, *HD* hemodialysis, *UFF* ultrafiltration failure

### Observation procedure

PD catheter removal was performed according to a previous report provided by the Department of Coloproctological Surgery in Juntendo University Hospital [14]. Under general anesthesia, a 3-cm skin incision was made at the opposite site of the PD catheter insertion to insert a versatile port (SILS® Port, Covidien, Japan) and then carbon dioxide was injected to achieve artificial pneumoperitoneum. An endoscope and 2 of the 5-mm ports were used during the observation and procedure in the peritoneal cavity.

### Semiquantitative morphological evaluation using laparoscopy (Fig. 1)

**Fig. 1:**
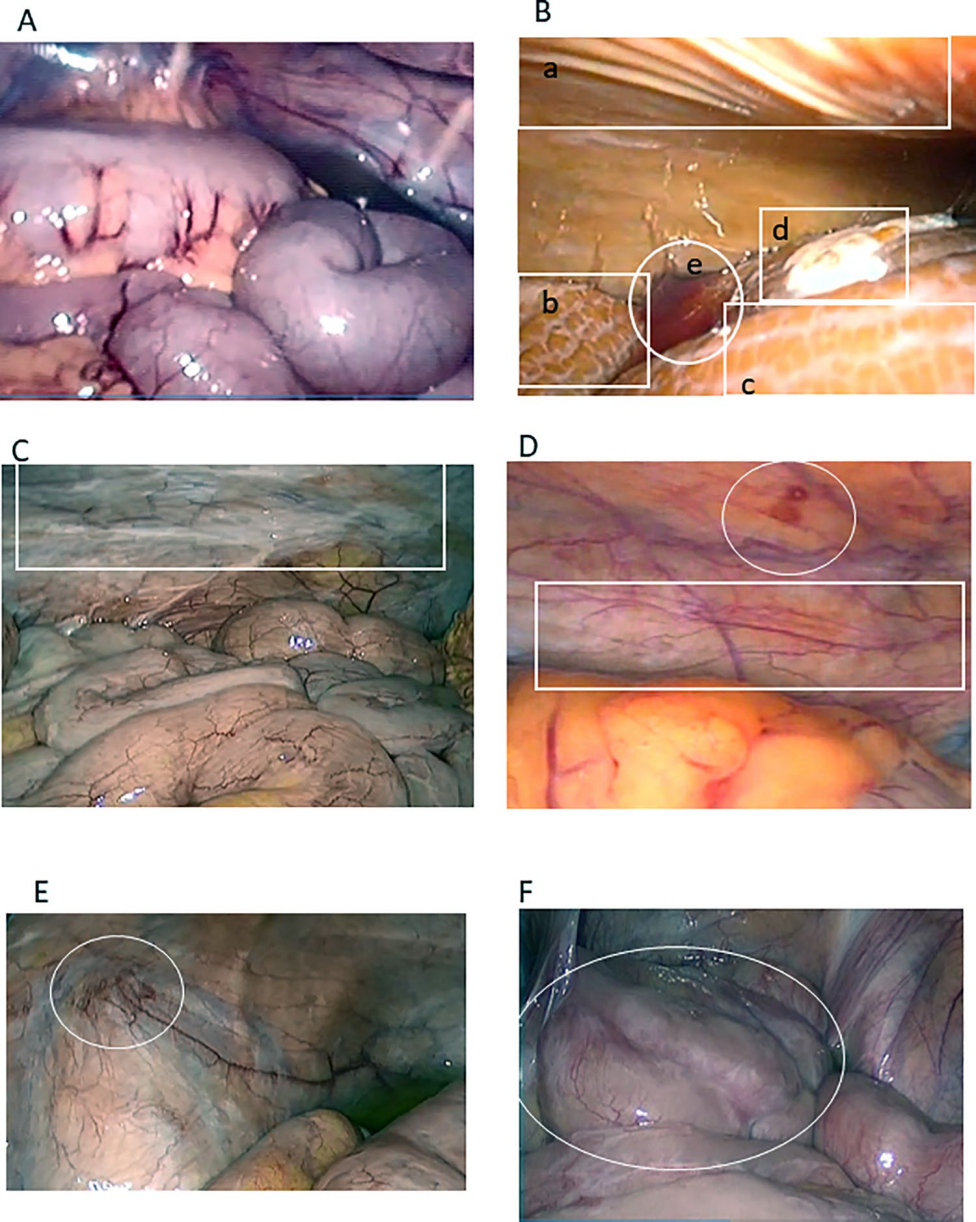
Characteristic laparoscopic findings of peritoneal damage. **A** Laparoscopic findings 2 months after the start at omental entanglement surgery. **B** Laparoscopic findings of patients with PD 10 years. The entire peritoneum is browning. a: crinkling changes in the parietal peritoneum. b: granular changes in the visceral peritoneum. c: cobblestone appearance on the surface of the intestinal tract. d: Massive fibrin deposit on the visceral peritoneum, e: bloody ascites. **C** Laparoscopic findings of patients with PD 5 years. Whitening change in parietal and visceral peritoneum. Vascular dilatation and tortuous. **D** Laparoscopic findings of patients with PD 4 years. Bleeding spots on the parietal peritoneum. Vascular dilatation. **E** Laparoscopic findings of patients with PD 5 years. Adhesion between the parietal wall and the intestine. Vascular spider appearance in parietal peritoneum. **F** Laparoscopic findings of patients with PD 5 years. Newly formed membrane

Based on our previous report [13], we assessed 4 major items of laparoscopic findings including changes in peritoneal surface color and properties, vascular findings, adhesion in both the parietal and visceral peritoneum, and others such as cloudiness of the liver surface, intestinal hypoperistalsis, and omentum atrophy. Peritoneal surface color changes were evaluated for whiteness, redness, and browning. Peritoneal surface properties were evaluated for fibrin deposition, newly formed membrane (NFM), granular appearance, crinkling, and cobblestone appearance. As vascular findings on the peritoneal surface, the presence or absence of vascular spider appearance, cirsoid-like appearance, dilatation, and tortuous was evaluated. Visceral and parietal adhesions were evaluated as adhesion. The observation points of the laparoscopic finding are 15 points in 4 sites in the abdominal cavity, and total 60 points are evaluated for each case.

### Observation site

These evaluated findings were first divided into 2 parts, the parietal side and the visceral side, and further divided into 4 parts (upper right part, right lower part, left upper part and left lower part). The presence or absence of these findings was converted into points, totaled for each item and scored. If there were evaluation findings in any of the 4 parts of the visceral or parietal peritoneum, 1 point was assigned to each peritoneal side. Total peritoneal surface color change score was 6 points, peritoneal surface property score was 10 points, vascular findings were 10 points, and adhesions were 4 points. Total laparoscopic score was 33 points (15 points in each visceral and parietal peritoneum and 3 other points).

### Peritoneal biopsy

Anterior parietal peritoneal samples from at least 5 cm of the site of original catheter insertion were obtained during catheter removal. The peritoneal samples were immediately fixed in formalin solution and then stained with hematoxylin and eosin, periodic acid–Schiff (PAS), Masson’s trichrome, or elastic van Gieson. Imaging System KS400 (Kortron Elektronik GmbH, Germany) was used for histological analysis.

### Microscopic finding evaluations

We evaluated SMC thickening and vascular patency in postcapillary venules as microscopic findings, based on previous reports [3]. SMC thickness was an average value of SMC thicknesses measured at 5 random points. Vascular patency calculated by diameter was shown as a diameter ratio, b/a (%) [4]. The vascular patency was the average value of vascular patency measured by 5 postcapillary venules.

### Statistical analysis

Data were expressed as mean ± standard deviation. Pearson’s correlation coefficient was used for the correlation between the 2 groups. Mann–Whitney U test was used to compare the clinical parameters between the 2 groups. *P* < 0.05 was considered statistically significant. JMP-J software program version 14.0 (SAS Institute Inc. Cary, NC, USA) was used for all statistical analyses.

## Results

Approval for laparoscopic observation was obtained for abdominal surgery in PD patients, and 95 cases had no cases of infectious diseases such as peritonitis one month before surgery. Of these, 78 were cases at the time of catheter removal where the abdominal cavity could be evaluated by video images. Of the 78 cases, approval was obtained for the collection of peritoneal tissue when the catheter was removed, and the number of evaluable samples was 45.

### Relationship between morphological findings and PD duration, serum levels of beta 2 microglobulin

**Fig. 2:**
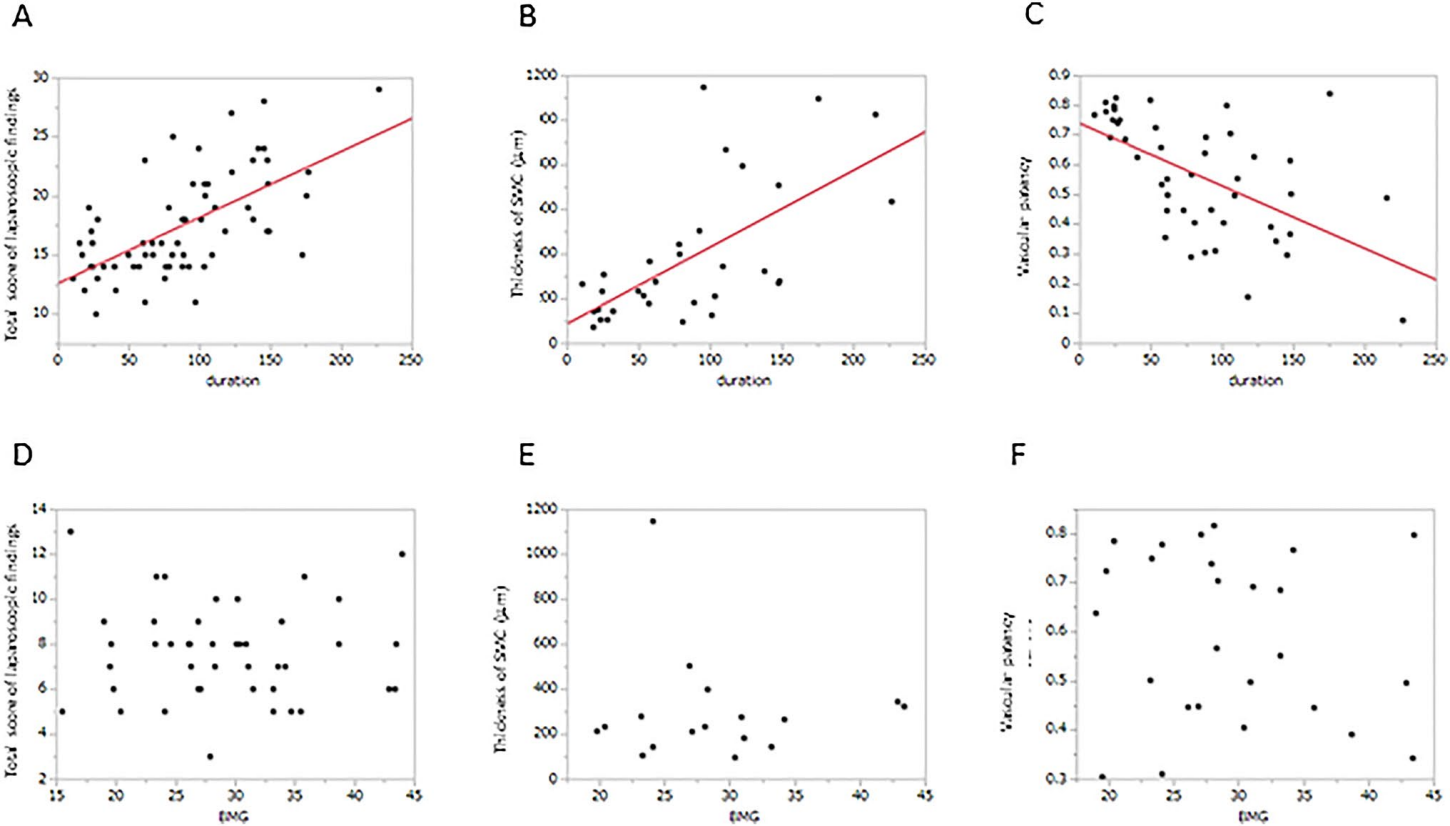
Relationship between morphological findings and PD duration. The total score of laparoscopic findings was significantly related to PD duration (*P* < .0001, **A**). Both average SMC thickness and patency calculated from the diameter had positive relationship with PD duration (*P* < 0.00001, **B** and **C**)

Total score of laparoscopic findings was significantly related to PD duration (*P* < 0.0001, Fig. 2A). Average SMC thickness showed a positive relationship with PD duration (*P* < 0.00001, Fig. 2B). The patency calculated from the diameter was also significantly related to PD duration (*P* < 0.0001, Fig. 2C). There was no relationship between serum level of beta 2 microglobulin and morphological findings (Fig. 2D–F).

### Relationship between laparoscopic findings’ score and SMC thickness and vascular patency

**Fig. 3:**
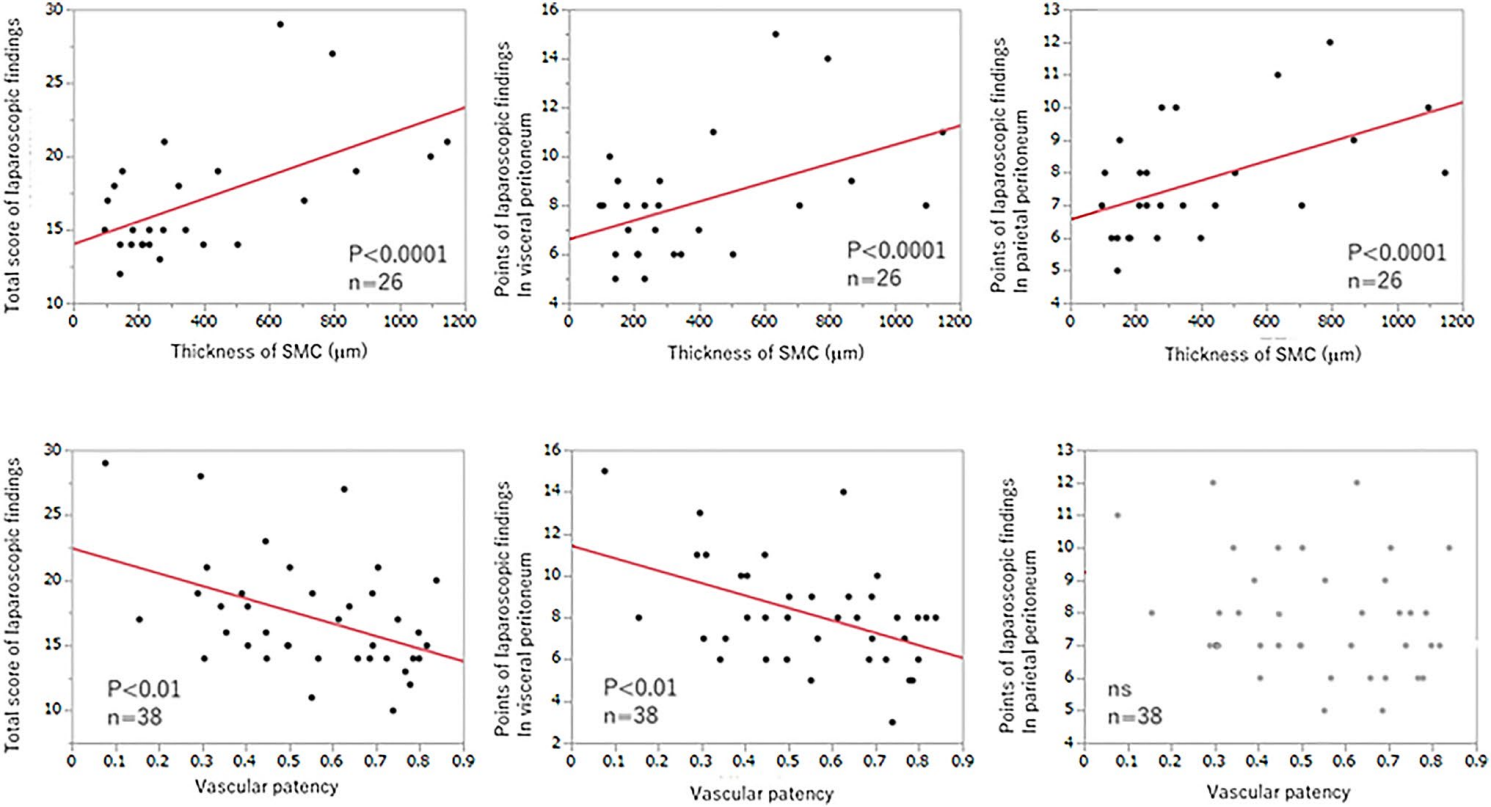
Relationship between laparoscopic score and microscopic findings. The total score of laparoscopic findings was related to SMC thickness (*P* < 0.0001, **A**). SMC thickness was related to both scores of laparoscopic findings in visceral and parietal peritoneums (*P* < .0001, **B** and **C**). The total score of laparoscopic findings had good relationship to vascular patency (*P* < .001, **D**). Vascular patency was related to the score of laparoscopic findings in visceral peritoneum (*P* < .001, **E**). There was no relationship between vascular patency and the of laparoscopic findings in parietal peritoneum (**F**)

Total scores of laparoscopic findings in both visceral and parietal peritoneum, in the visceral peritoneum, and in the parietal peritoneum were significantly related to the average SMC thickness (*P* < 0.0001, Fig. 3A–C). Total scores of laparoscopic findings in the whole peritoneum and in the visceral peritoneum were significantly related to vascular patency (*P* < 0.01, Fig. 3D and E). The points of laparoscopic findings in the parietal peritoneum did not significantly relate to vascular patency (Fig. 3F).

### Relationship between microscopic findings and individual laparoscopic finding: changes in peritoneal surface color, adhesions, changes in peritoneal surface property, and changes in vascular findings

As the scores of peritoneal surface color changes, peritoneal surface structural findings, and vascular findings among the observed laparoscopic findings were scattered among patients, we examined the relationship between the score of these findings and SMC thickness and vascular patency.

**Fig. 4:**
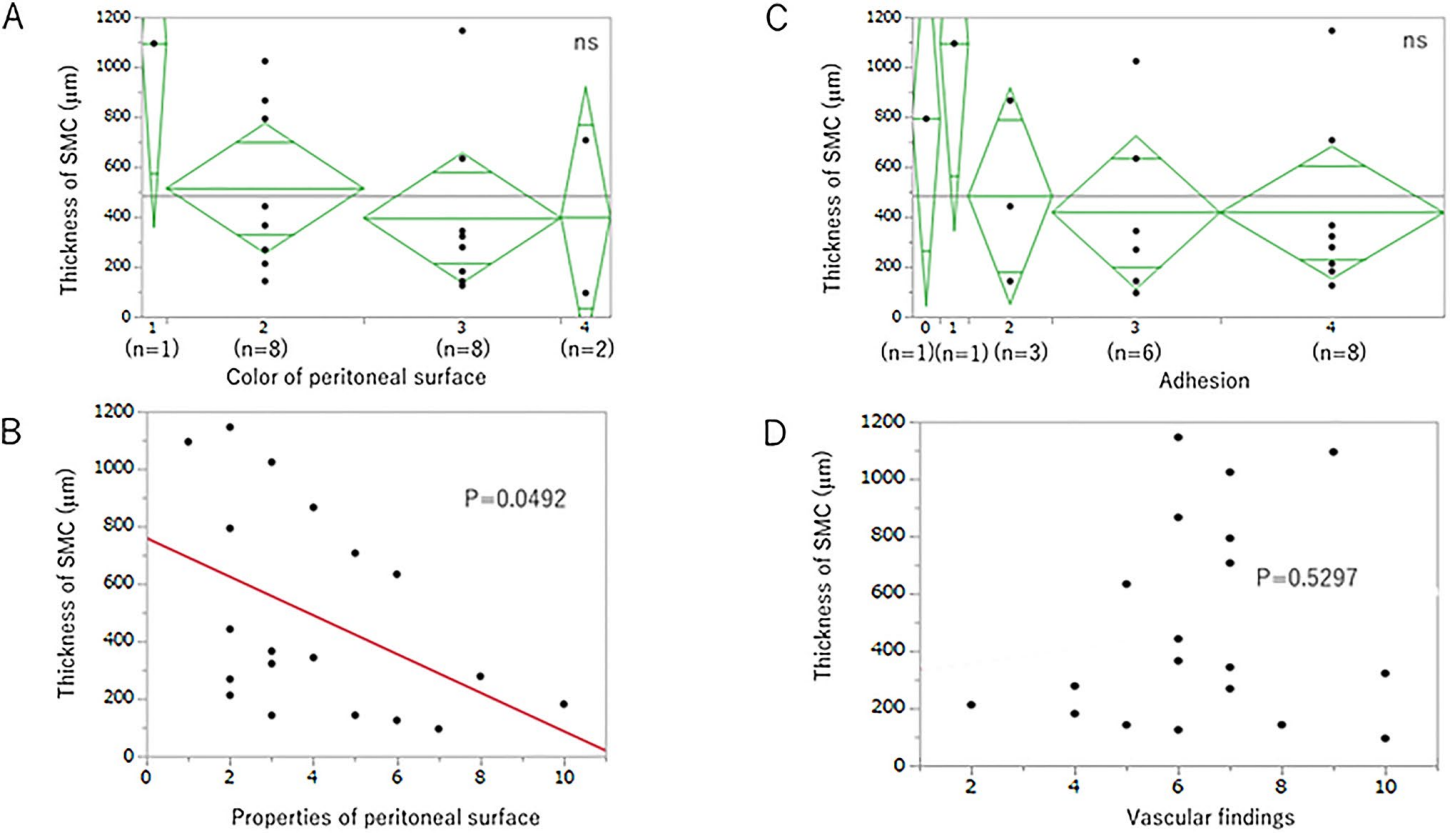
Relationship between SMC thickness and individual laparoscopic finding: changes in peritoneal surface color, adhesions, changes in peritoneal surface properties, and changes in vascular findings. No significant difference was found in average SMC thickness among all groups (**A**). The score of peritoneal surface properties was related to SMC thickness (*P* = .0492, **B**). No significant difference was found in average SMC thickness among all groups (**C**). The score of vascular findings was not related to SMC thickness (*P* = .6767, **D**)

The score of peritoneal surface color changes was divided into 4 groups: 1, 2, 3, and 4 points. There were 19 participants for whom comparison of SMC thickness and peritoneal surface color changes was possible (1, 8, 8, and 2 participants in groups 1, 2, 3, and 4, respectively). No significant difference was found in the average SMC thickness among all groups (Fig. 4A). The score of peritoneal surface properties was related to SMC thickness (*P* < 0.05, Fig. 4B). The score of adhesion was divided into 5 groups: 0, 1, 2, 3, and 4 points. There were 19 participants for whom comparison of SMC thickness and adhesion score was possible (1, 1, 3, 6, and 8 participants in groups 0, 1, 2, 3, and 4, respectively). No significant difference was found in SMC thickness among all groups (Fig. 4C). The score of vascular findings was not related to SMC thickness (Fig. 4D).

**Fig. 5:**
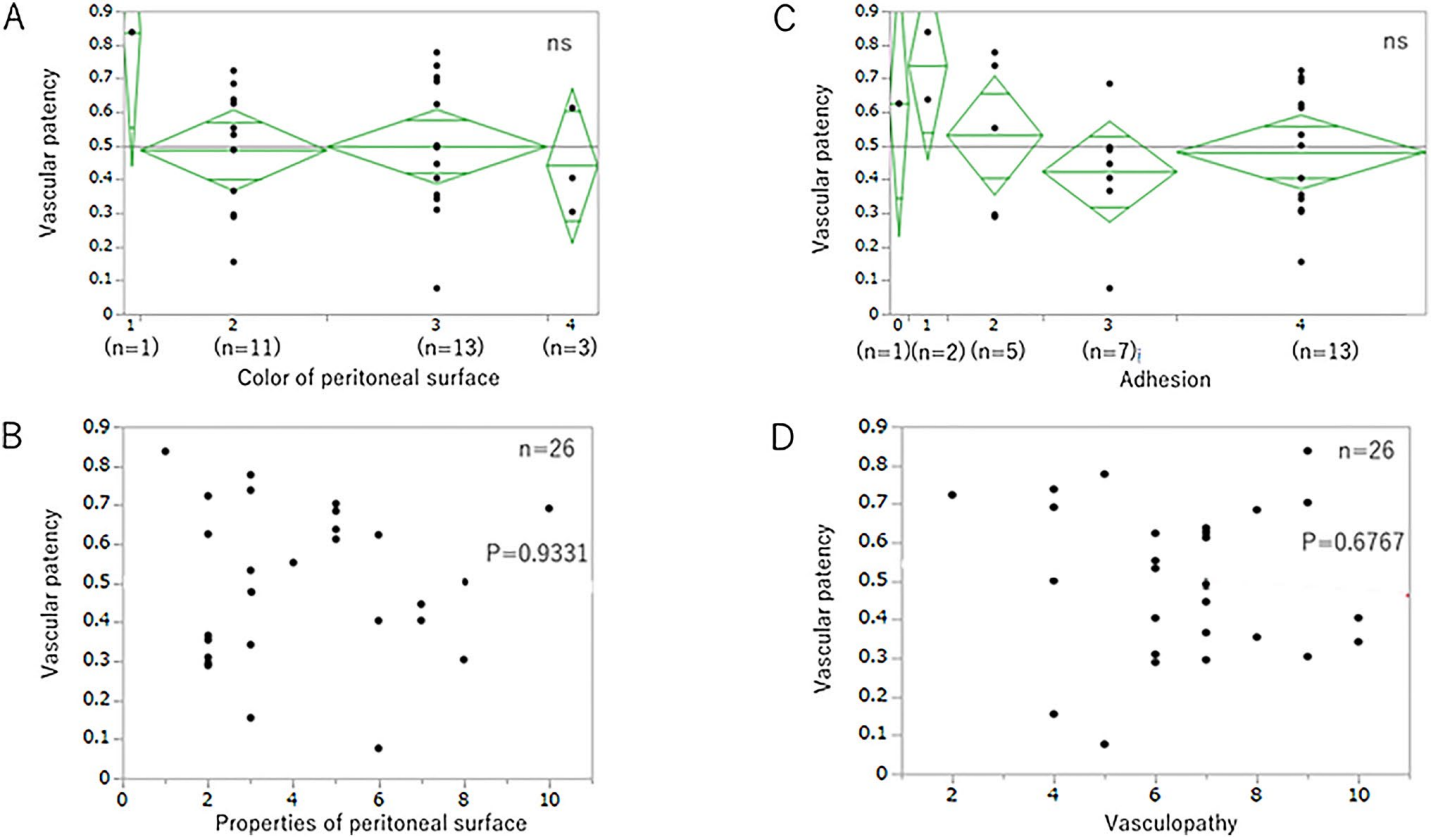
Vascular patency and individual laparoscopic finding: changes in peritoneal surface color, adhesions, changes in peritoneal surface properties, and changes in vascular findings. No significant difference was found in vascular patency among all groups (**A**). The score of peritoneal surface properties was not related to vascular patency (*P* = .0492, **B**). No significant difference was found in vascular patency among all groups (**C**). The score of vascular findings was not related to vascular patency (**D**)

There were 28 participants for whom comparison of vascular patency and color change was possible (1, 11, 13, and 3 participants in groups 1, 2, 3, and 4, respectively). No significant difference was found in vascular patency among all groups (Fig. 5A). The score of peritoneal surface properties was not related to vascular patency (Fig. 5B). There were 28 participants for whom comparison of vascular patency and adhesion score was possible (1, 2, 5, 7, and 13 participants in groups 0, 1, 2, 3, and 4, respectively). No significant difference was found in vascular patency among all groups (Fig. 5C). The score of vascular findings was not related to SMC thickness (Fig. 5D).

### Laparoscopic score and microscopic findings in PD patients with NFM, intestinal hypoperistalsis, and omentum atrophy

**Fig. 6:**
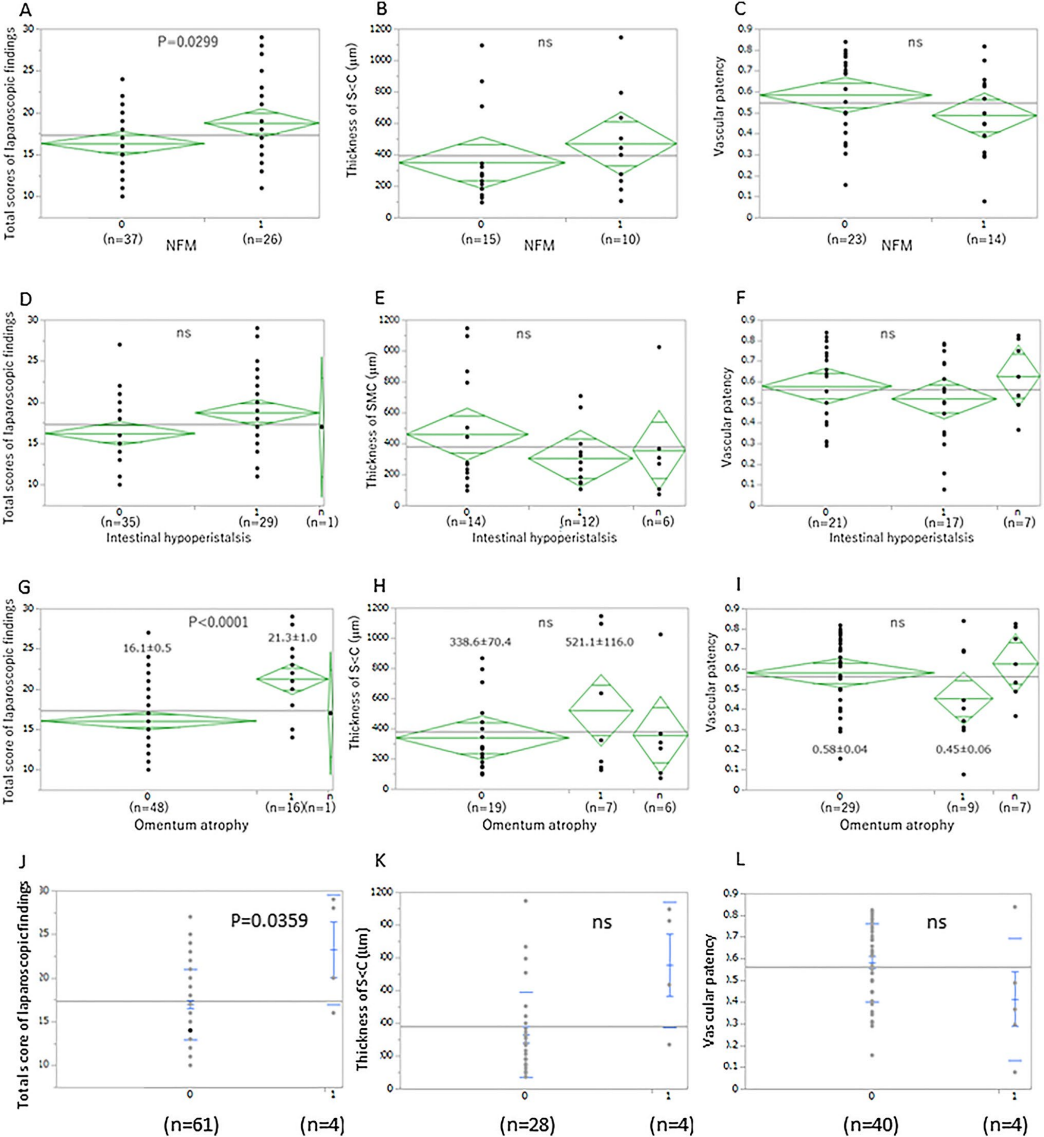
Laparoscopic score and microscopic findings in PD patients with newly formed membrane, intestinal hypoperistalsis, and omentum atrophy. Laparoscopic score was significantly higher in patients with a newly formed membrane compared with those without (*P* = .00299, **A**). There was no significant difference in SCM thickness nor vascular patency (**B** and **C**). There was no morphological difference between patients with intestinal hypoperistalsis (**D**–**F**). The laparoscopic score in patients with omentum atrophy was significantly higher than that those without (*P* < .0001, **G**). There was no difference in SCM thickness nor vascular patency between the 2 groups (**H** and **I**). Laparoscopic scores were significantly higher in EPS patients compared with non-EPS patients (*P* < .05, **J**). Neither SMC thickness nor vascular patency showed statistically significant difference between the 2 groups (**K** and **L**)

As clinical findings of EPS are intestinal peristalsis, ileus, and tissue sclerotic findings, we examined differences of laparoscopic and microscopic findings between patients with and without NFM, intestinal hypoperistalsis, and omentum atrophy. Laparoscopic scores in patients with NFM were significantly higher (*P* < 0.05, Fig. 6A) compared with patients without, but there was no difference in SCM thickness nor vascular patency (Fig. 6B and C). There was no morphological difference in patients with intestinal hypoperistalsis (Fig. 6D–F). The laparoscopic score in patients with omentum atrophy was significantly higher compared with patients without (*P* < 0.0001, Fig. 6G), but there was no difference in SCM thickness nor vascular patency between them (Fig. 6H and I).

### Morphological changes in patients with EPS

We examined the morphological findings at the time of PD catheter removal in patients who developed EPS after PD discontinuation. Laparoscopic scores were significantly higher in EPS patients compared with non-EPS patients (*P* < 0.05, Fig. 6J). In the EPS group, the color tone points in the visceral peritoneum (3 points in total) were 2.25 (1.4 in the non-EPS group), and whitening and browning were observed in all cases. The surface evaluation of the visceral peritoneum (5 points in total) was 3.25 in the EPS group and 1.48 in the non-EPS group, and cobblestones and granular appearances were observed in all cases. SMC thickness tended to be higher in EPS group, but did not show a statistically significant difference (Fig. 6K). Similarly, vascular patency also tended to be low in EPS group, but did not show a statistically significant difference (Fig. 6L).

## Discussion

This study revealed that the total laparoscopic finding’s score (LFS) and microscopic findings, SMC thickness and vascular patency, were related with PD duration. Total LFS was related to SMC thickness in both visceral and parietal peritoneum, whereas total LFS was related to vascular patency in parietal but not visceral peritoneum. There was no relationship between microscopic findings and peritoneal surface color, properties, vasculopathy, and adhesion. Total LFS was significantly higher in patients with NFM and omentum atrophy compared with those without.

Macroscopic observations of the peritoneal cavity using a laparoscope have been performed during operations to manage conditions such as catheter infection, drainage failure, and catheter removal for PD discontinuation [14–16]. Laparoscopic observation was possible to confirm causes of PD catheter occlusion, adhesion between the omentum, intestinal tract, and the inner cuff, and a partial intestine adhesion by NFM that cannot be detected by computed tomography [15, 17–19]. In our previous report, we examined macroscopic morphological findings such as fibrin deposition, peritoneal membrane opacity, vascular structure, and presence of adhesion and calcification in both parietal and visceral peritoneums of the upper and lower peritoneal cavities and semiquantitatively determined the scores [13]. The study revealed that the total macroscopic score increased with PD duration. Peritoneal membrane opacity, fibrin deposition, and calcification were observed in the entire peritoneal cavity. The scores of fibrin deposition, opacity, and calcification increased with PD duration. Vascular network changes in the parietal peritoneum were more serious compared with those in the visceral peritoneum, but there was no difference in the vascular network changes between the upper and lower areas [13].

Pathological alterations are characterized by the disappearance of mesothelial cells, fibrosis, and thickening of the mesothelial interstitium, thickening of the walls, and stenosis/occlusion of the lumen of small blood vessels, angiogenesis, and lymphatic vessel proliferation [20, 21]. Williams et al. [5] in 2002 and Honda et al. [3] in 2008 proposed the quantification (semiquantification) method of the histological characteristics of the peritoneum of patients undergoing PD. They proposed that uremic condition and PD duration play important roles in the development of peritoneal deterioration.

Since this study presented that LFS increased along with PD period and pathological findings such as SMC thickening and decreased vascular patency, laparoscopic findings were also confirmed to be an evaluation method that reflects the morphological changes of the peritoneum due to continued PD. Furthermore, a statistical correlation was found between LFS and SMC thickness and vascular patency and confirmed the relevance of the evaluation of morphological peritoneal injury of pathological and macroscopic findings in PD patients.

A macroscopic feature found in patients with EPS is a white film-like structure, consistent with an NFM on the surface of the visceral peritoneum [22]. Nakamoto et al. [23] presented a classification of EPS that introduced the following 4 stages based on clinical and imaging findings using plain abdominal radiographs, contrast imaging, ultrasound, computed tomography, and magnetic resonance imaging. The first stage is a pre-EPS stage without the development of a newly formed capsule, with fibrin exudation/bleeding due to sustained inflammation/exudation reaction. The second stage is an EPS-I stage (cystic stage) observed in the capsule where exudate fibrin accumulates in the existing peritoneum and fibrin capsules. The third stage is the EPS-II stage (adhesion stage), as an encapsulating stage (characterized by ileus, mild to severe inflammation, whole encapsulation, and intestinal adhesion). The fourth and final stage is the EPS-III stage (scar stage). Laparoscopic observation is possible to confirm a partial intestine adhesion by NFM that cannot be detected by computer tomography, and early interventions have been available even for patients with mild clinical symptoms of EPS according to our experience [19]. However, characteristic peritoneal tissue injury that can predict the onset of EPS after PD discontinuation has not yet been determined.

There have been several reports of the relationship between pathological and clinical findings in PD patients [1, 3, 8, 24]. However, pathological findings provide information for a very limited part of the abdominal cavity and are not an assessment of the visceral peritoneum, which is the dominant clinical manifestation of EPS. In this study, we examined the relevance of clinical findings, including pathological findings, and laparoscopic findings to clarify the characteristics of macroscopic findings in PD patients and tried to examine the characteristics of laparoscopic findings in predicting progression to EPS. Only LFS in patients with NFM was significantly higher than those without. Of course, the number of EPS patients was too small to suggest characteristic findings, but laparoscopic findings are clearly associated with pathological findings, suggesting that laparoscopic findings may involve more histological changes in the injured peritoneum of PD patients. There remained several problems to be solved in macroscopic observations by laparoscopy. For instance, severe opacity in the peritoneum makes it difficult to evaluate vascular findings. Extensive adhesion narrows observable area. It is desirable to extract specialized findings in peritoneal injury due to PD based on pathophysiology and to establish an evaluation method that reflects the severity of injury.

## Conclusion

There was a significant relationship between microscopic and laparoscopic findings in PD patients. It is suggested that SMC thickening and vascular stenosis were related to macroscopic peritoneal injury. It is important to evaluate laparoscopic findings in more PD patients to find predictive findings for EPS development.
